# Supplementary material for: Risk and resilience factors in problematic internet use among Sámi and non-Sámi adolescents in Finnmark, Arctic Norway: The role of social norms and ethnic identity
Source: PLoS One. 2026 Aug 26;21(8):e0356920. doi: 10.1371/journal.pone.0356920 (PMC13513970; doi:10.1371/journal.pone.0356920)
Supplement: Supplementary file 1 [file pone.0356920.s001.pdf]

Author Response to Requested Revisions

Shiho Hansen
Sami Norwegian National Advisory
Unit for Mental Health and Substance
Use, Finnmark Hospital Trust
Stuorraluohkka 34, 9730 Karasjok,
Norway

September 23, 2025
Academic Editors
PLOS ONE

Dear Academic Editors,

I am writing in response to the email *"PLOS ONE: Edits requested on your submission PONE-D-25-35264 - [EMID:477c279eefa09786]."*

When I initially submitted the first version of this manuscript, the ethics approval process was still ongoing. Unfortunately, I was unable to obtain a response from the IRB before the resubmission deadline. In light of this, I decided to withdraw that initial submission and prepare a new manuscript only after the IRB review process was fully completed.

In response to the comments provided in the email:

1. *Please upload a copy of your study protocol that was approved by your ethics committee/IRB as a Supporting Information file. By the study protocol, we mean the complete and detailed plan for the conduct and analysis of the trial approved by the ethics committee/IRB. Please send this in the original language. If this is in a language other than English, please also provide a translation.*

   Response: After obtaining ethics approval for this study from the IRB of my institution, I uploaded the original study protocol in Norwegian as well as an English translation as file type "Other." Additionally, the IRB document number has been added in the *Ethics and Data Protection* section of the Methods (p. 19, l. 10).

2. *Please complete all items on the Human Participants Research Checklist that are relevant for your submission, by following this link (link is omitted because of its length). There may be overlap between the checklist items and other queries listed below; please address any duplicated queries both in your response email and on the checklist itself. Upload the completed Human Participants Research Checklist as file type "Other" when you re-submit your manuscript. This document is for internal journal use only and will not be published if your article is accepted. The requested information will help us to assess whether your submission complies with PLOS ONE's policies and adheres to applicable reporting standards.*

Response: I have completed the Human Participants Research Checklist in full and uploaded it as file type “Other.”

3. *Please provide additional details regarding participant consent. In the ethics statement in the Methods and online submission information, please ensure that you have specified what type you obtained (for instance, written or verbal, and if verbal, how it was documented and witnessed). If your study included minors, state whether you obtained consent from parents or guardians. If the need for consent was waived by the ethics committee, please include this information.*

   Response: Thank you for this comment. I have expanded the description of participant consent in the Methods section as follows: “All participants will be over 16 years of age and will provide their own informed consent in accordance with Norwegian regulations. Consent will be obtained either in written (paper) or digital form. Participants will indicate their agreement by checking the consent declaration form without providing names or other personal identifiers, as the survey will be conducted anonymously. Study information and consent forms will be made available in both Norwegian and Northern Sámi. No minors will be included in the study, and therefore parental or guardian consent is not required.” (p. 19, l. 17 – p. 20, l. 4).

3.1. *Once you have amended this/these statement(s) in the Methods section of the manuscript, please add the same text to the “Ethics Statement” field of the* submission *form (via “Edit Submission”).*

   Response: I have also uploaded the same text to the “Ethics Statement” field of the submission form (via “Edit Submission”).

I hope this resolves the concern, and I sincerely appreciate your understanding. Please let me know if any additional documentation or clarification is required.

Please address all correspondence regarding this manuscript to me at shiho.hansen@finnmarkssykehuset.no.

Thank you for considering this manuscript.

Sincerely,

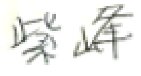

Shiho Hansen
