# Supplementary material for: Risk and resilience factors in problematic internet use among Sámi and non-Sámi adolescents in Finnmark, Arctic Norway: The role of social norms and ethnic identity
Source: PLoS One. 2026 Aug 26;21(8):e0356920. doi: 10.1371/journal.pone.0356920 (PMC13513970; doi:10.1371/journal.pone.0356920)
Supplement: Supplementary file 2 [file pone.0356920.s002.docx]

**Response to the Reviewers’ Comments**

[**Editor’s comments**	1](#_Toc230616329)

[**Reviewers' Comments to the Author**	3](#_Toc230616330)

[**Reviewer 1**:	3](#_Toc230616331)

[**Minor comments**	10](#_Toc230616332)

[**Reviewer 2:**	12](#_Toc230616333)

[**Minor Revisions:**	16](#_Toc230616334)

[**Other revisions:**	21](#_Toc230616335)

# **Editor’s comments**

**Comment 1:**

*Journal requirements:*

*When submitting your revision, we need you to address these additional requirements.*

*1. Please ensure that your manuscript meets PLOS ONE's style requirements, including those for file naming. The PLOS ONE style templates can be found at*

[*https://journals.plos.org/plosone/s/file?id=wjVg/PLOSOne_formatting_sample_main_body.pdf*](https://journals.plos.org/plosone/s/file?id=wjVg/PLOSOne_formatting_sample_main_body.pdf) *and*

[*https://journals.plos.org/plosone/s/file?id=ba62/PLOSOne_formatting_sample_title_authors_affiliations.pdf*](https://journals.plos.org/plosone/s/file?id=ba62/PLOSOne_formatting_sample_title_authors_affiliations.pdf)

**Response:**

We thank the editorial office for the additional guidance. The manuscript has been fully revised to comply with PLOS ONE formatting and style guidelines, including use of the official templates and correct file naming conventions.

**Comment 2:**

*Kindly amend the title of your manuscript to include the word "protocol" in it.*

**Response:**

We thank the editorial office for the helpful suggestion. “Study Protocol” has been added at the beginning of the title (p. 1, l. 4).

**Comment 3:**

*Thank you for stating the following in the Acknowledgments Section of your manuscript:*

*“We gratefully acknowledge the support of the Research and Development Fund of the Sámi Norwegian National Advisory Unit for Mental Health and Substance Use (SANKS), which enabled the preparation of this research protocol. We also thank the young people who participated in the participatory meetings for their valuable contributions to the development of the questionnaire. We further acknowledge Ronald Isaksen, Administrative Coordinator at SANKS, for his role in organizing and facilitating meetings with youth and schools. Finally, we appreciate the Research Department at Finnmark Hospital Trust for providing a supportive and enabling research environment.”*

*We note that you have provided funding information that is currently declared in your Funding Statement. However, funding information should not appear in the Acknowledgments section or other areas of your manuscript. We will only publish funding information present in the Funding Statement section of the online submission form.*

*Please remove any funding-related text from the manuscript and let us know how you would like to update your Funding Statement. Currently, your Funding Statement reads as follows:*

*“The development of the study protocol was supported by the Research and Development Fund of the Sámi Norwegian National Advisory Unit for Mental Health and Substance Use (SANKS), Finnmark Hospital Trust. The funders had no role in the analysis or interpretation of data, nor did they have any authority over the writing or publication of the manuscript. No additional external funding was received for this study. Funding for the study itself will primarily be provided by Helse Nord, supplemented by institutional research funds from SANKS and Finnmark Hospital Trust. Publication fees and indirect costs will be covered by the hosting institutions.”*

*Please include your amended statements within your cover letter; we will change the online submission form on your behalf.*

**Response:**
Thank you for your helpful guidance. We have removed all funding-related text from the Acknowledgements section of the manuscript (p. 32), in accordance with your instructions. We confirm that no funding information now appears in any section of the manuscript.

We have revised the Funding Statement accordingly in the online submission form. In doing so, we would like to clarify that the Research and Development Fund of the Sámi Norwegian National Advisory Unit for Mental Health and Substance Use (SANKS) is not an internal institutional fund. Although it is administered by SANKS, it is open to external applicants and involves a competitive review process. The fund is therefore independent of the authors’ institutional affiliation.

As requested, the funding information has been removed from the manuscript and is now provided only in the Funding Statement section of the online submission form. The amended wording has also been included in this cover letter. We kindly ask that the submission system be updated accordingly.

# **Reviewers' Comments to the Author**

## **Reviewer 1**:

*This is a clearly written and thoughtfully designed protocol focusing on PIU and positive internet use among Sámi and non-Sámi adolescents in Finnmark. The integration of social norms theory and ethnic identity constructs is well articulated, and the cultural and ethical considerations for Indigenous research are carefully addressed.

Below are several major and minor comments that I believe will strengthen the manuscript and improve its clarity and reproducibility.*

**Major comments**
**Comment 1:** *Clarify the operationalization of PIU and positive internet use. You state that PIU will be measured using the Problematic Internet Use Questionnaire (PIUQ), and that positive internet use will be captured via items reflecting constructive, identity-supporting, and socially connective internet activities. Please specify explicitly:

(i) Whether the primary PIU outcome in the PLS-SEM models will be a latent factor, a total scale score, or both.
(ii) Whether you plan to use any clinical or empirical cut-off to define “problematic” vs. “non-problematic” internet use (even if only for descriptive purposes), or if PIU will be treated purely as a continuous construct.
(iii) How you will operationalize positive internet use in the structural models (e.g., separate latent factor, composite score, or multiple observed indicators).*

**Response:**

Thank you for your comment. Below, we provide a revised clarification regarding the operationalization of Problematic Internet Use (PIU) and positive internet use, specifically addressing the use of the PIUQ-9 in our study.

(i) Primary PIU Outcome (Latent vs. Total Score)

In the Partial Least Squares Structural Equation Modeling (PLS-SEM) framework, PIU will be modeled as a latent construct with three reflective dimensions: obsession, neglect, and control disorder, as defined by the Problematic Internet Use Questionnaire, Short Form (PIUQ-9). These dimensions are well-supported by the psychometric structure of the PIUQ-9 (Koronczai et al., 2011; Laconi et al., 2019).

For descriptive purposes, we will also calculate and report the total PIUQ-9 score (sum of all 9 items, ranging from 9 to 45). This dual approach allows us to leverage the strengths of both latent variable modeling (for hypothesis testing) and total score reporting (for descriptive comparisons).

This response has been incorporated into measurement of PIU (p.17, l.17–p.18, l.3) in WP1.1: Measuring instruments and Descriptive statistics (p.23, l.3–10) in the Data analysis section.

(ii) Cut-Off vs. Continuous Treatment of PIU

1. Modeling Approach:
   In all structural models, PIU will be treated as a continuous latent variable. This approach ensures that the full range of variability in PIU is captured, aligning with the psychometric properties of the PIUQ-9.
2. Descriptive Prevalence Reporting:
   For descriptive purposes, we will apply a pre-registered cut-off score of ≥ 22 for the PIUQ-9 (total range: 9–45). This threshold is based on proportional scaling from the original PIUQ-18 cut-off (≥ 41) and aligns with prior psychometric recommendations (Koronczai et al., 2011). While the PIUQ-9 has been validated across diverse cultural contexts, no universally accepted diagnostic cut-off exists (Laconi et al., 2019; Burkauskas et al., 2020). Therefore, the cut-off of ≥ 22 will serve as a descriptive benchmark rather than a diagnostic criterion.

These responses have been reflected into the measurement of PIU (p.18, l.4-13) in WP1.1: Measuring instruments, Descriptive statistics (p.23, l.3–10) and PLS-SEM (p.23, l.12-14) in Data analysis.

(iii) Positive internet use will be operationalized as a separate latent construct in the structural models, as mentioned in the manuscript (maintaining social connections, seeking social support and emotional connection, and engaging with cultural identity). We will conduct a pilot exploratory factor analysis (EFA) during the pretesting phase to confirm the factor structure. If the items load onto a single factor, they will be used as indicators of a unidimensional latent construct. If a multidimensional structure emerges, we will model positive internet use as a higher-order latent construct. Using EFA in the pilot phase ensures the construct is valid and reliable before inclusion in the main study.

This response has been incorporated into the measurement of Positive Internet Use in WP1.1: Measuring instruments (p.18, l.19–p.19, l.6) and PLS-SEM (p.23, l.14-16) in Data analysis.

References

- Koronczai, B., et al. (2011). Confirmation of the three-factor model of problematic internet use on offline adolescent and adult samples. *Cyberpsychology, Behavior, and Social Networking, 14*(11), 657–664.
- Laconi, S., et al. (2019). Psychometric evaluation of the nine-item Problematic Internet Use Questionnaire (PIUQ-9) in nine European samples of internet users. *Frontiers in Psychiatry, 10*, 136.
- Burkauskas, J., et al. (2020). Problematic internet use and its relationship with health-related quality of life in a cross-sectional sample: Mediating role of behavioral avoidance. *International Journal of Environmental Research and Public Health, 17*(22), 8617.

**Comment 2:**

*In your Introduction, you correctly note that PIU is associated with mental health difficulties and some health-risk behaviors, and you reference prior work on PIU in adolescents and young adults. To further strengthen this section, I recommend adding a large epidemiologic study that examined both PIU-substance misuse associations and race/ethnicity-specific prevalence, including Indigenous college students:

Qeadan F, Egbert J, English K. (2022). “Associations between problematic internet use and substance misuse among US college students.” Computers in Human Behavior, 134, 107327.

This study uses data from over 400,000 U.S. college students to quantify the associations between PIU and multiple forms of substance misuse and substance use disorder, and report PIU prevalence across demographic subgroups, including a specific race/ethnicity category for Indigenous students (“NH AI/AN/NH” in Table 1). Including this reference would not only situate your work within the broader PIU-substance use literature for youth and young adults, but also provide readers with complementary prevalence estimates of PIU among Indigenous college students in another national context, which aligns closely with your focus on Sámi adolescents.*

**Response:**

Thank you for this constructive suggestion. We have incorporated the recommended large epidemiologic study by Qeadan, Egbert, and English (2022) into the Introduction under Internet use and Indigenous adolescents (p. 6, l.9-12), and we have added the full citation to the reference list. This addition strengthens our epidemiologic framing of PIU in youth/young‑adult populations and provides a relevant Indigenous comparison that aligns with our focus on Sámi adolescents.

**Comment 3:**

*The protocol defines Sámi identity based on both objective (ethnic background, language use, ancestry) and subjective (self-identification) criteria, and classifies participants as Sámi if they meet either objective or subjective criteria (or both). Non-Sámi participants are those who report no Sámi ethnic affiliation, language ties, or self-identification as Sámi. Given that your primary analyses will compare Sámi and non-Sámi groups via PLS-MGA, please clarify whether you will:

(i) Distinguish participants who meet only objective criteria from those who meet only subjective criteria, at least in descriptive statistics; and
(ii) Consider sensitivity analyses (e.g., restricting the Sámi group to those meeting both objective and subjective criteria) to assess robustness of any group differences.

Even if your primary analysis retains a single Sámi category, specifying these planned descriptive and sensitivity analyses would help readers appreciate the complexity of Indigenous identity and the potential heterogeneity within the Sámi group.*

**Response:**

We thank the reviewer for raising these important questions regarding the operationalization of Sámi ethnicity. Our approach has been further clarified and refined in response to both the reviewer’s comments and co-author feedback.

In this study, Sámi affiliation is classified using a combination of language use and self-identification, which serve as classification criteria rather than constructs under investigation. This approach is consistent with established practice in Sámi epidemiological and psychosocial research in Norway and has been widely applied in previous large-scale studies, including the North Norwegian Youth Study (NNYS), the Norwegian Arctic Adolescent Health Study (NAAHS), and Ungdata. It is also consistent with broader epidemiological definitions of ethnicity, which conceptualize ethnicity as a multifaceted construct based on “a mix of cultural and other factors including language, ancestry, and self-identity,” and recommend that researchers classify ethnic groups using one or a combination of these indicators (Bhopal, 2004). Using both objective indicators (e.g., language use) and subjective self-identification therefore provides a more comprehensive and theoretically grounded operationalization of Sámi ethnicity.

In addition, to enhance conceptual precision and avoid ambiguity, we have avoided the use of the terms “objective” and “subjective” and instead use the more specific descriptors “language use” and “self-identification,” consistent with established practice in Sámi research (Kvernmo & Heyerdahl, 2004). While the terms “objective” (e.g., ancestry or language use) and “subjective” (self-identification) have been used in some Sámi studies, we follow Kvernmo’s approach by using the descriptors “language use” and “self-identification.” This approach provides clearer and more precise descriptions of the classification criteria applied in the present study.

This combined approach is particularly important in the Norwegian context due to the long history of assimilation policies, which has resulted in discrepancies between ancestry, language use, and self-identification. As shown in previous research (e.g., Kvernmo et al., 2003), a substantial proportion of adolescents with Sámi background may not report Sámi self-identification. Relying solely on one type of indicator would therefore risk systematic misclassification. The primary aim of this study is not to develop new classification methods, but to apply established and comparable criteria to enable robust analysis of group differences.

This terminology has been applied consistently throughout the manuscript (Abstract, p.3; WP1.1: Classification of Sámi and non-Sámi adolescents in WP1.1 Measuring instruments (p.16-17); and PLS Multi Group Analysis (PLS-MGA) in WP3. Data analysis, p.24).

At the same time, we acknowledge that ethnic identity is a multidimensional and dynamic construct, and that no single classification approach can fully capture its complexity. This limitation is explicitly discussed in the revised manuscript (Discussion section, p. 31, l.6-9).

**Clarification of group composition**

Additionally, we have added a clarification of the group composition to improve transparency:

- The non-Sámi group consists primarily of majority Norwegian adolescents and ethnocultural Kven youth, reflecting the demographic composition of Finnmark. Adolescents from other minority backgrounds are included in this group but are not analyzed separately due to small numbers.
- Adolescents classified as Sámi include individuals with both mono-ethnic and multi-ethnic Sámi backgrounds.

This revision has been incorporated into the section Classification of Sámi and non-Sámi adolescents in WP1.1 Measuring (p.17, l. 8-13).

(i) Distinguishing objective‑only, subjective‑only, and combined Sámi identifiers in descriptive statistics

In response to the reviewer’s suggestion, we will provide descriptive statistics differentiating adolescents who meet only language-based criteria, only self-identification criteria, or both. This will allow readers to clearly see the heterogeneity within the Sámi group, which is particularly important in light of assimilation processes that have created discrepancies between ancestry and self‑identification.

This revision is now reflected in Classification of Sámi and non-Sámi adolescents in WP1.1 Measuring instruments (p.16-17) and in Descriptive statistics (p.23, l.3–10) within the Data analysis.

(ii) Conducting sensitivity analyses restricting the Sámi group to participants meeting both both language-use and self-identification criteria

We will also conduct sensitivity analyses restricting the Sámi group to adolescents who meet both language-use and self-identification criteria. This will allow us to evaluate the robustness of PLS‑MGA findings under a more conservative classification strategy. Given the known divergence between ancestry and self‑identification among Sámi youth (e.g., Kvernmo et al., 2003), such sensitivity analyses will help determine whether group differences are stable across identity definitions influenced by historical and sociopolitical conditions.

This revision was reflected in PLS Multi Group Analysis (PLS-MGA) in WP3. Data analysis, p.24, l.17-21).

References

- Kvernmo, S., Heyerdahl, S., & Nordahl, H. M. (2003). *Ung i Sápmi. Helse, identitet og levekår blant samisk ungdom* [Young in Sápmi. Health, identity, and well-being among Sámi adolescents]. Centre for Sámi Health Research, University of Tromsø.
- Bhopal, R. (2004). Glossary of terms relating to ethnicity and race: For reflection and debate. *Journal of Epidemiology and Community Health, 58*(6), 441–445.

**Comment 4:**

*You plan to use PLS-SEM to model the relationships among social norms, ethnic identity, ethnic victimization, and PIU/positive internet use, and to employ PLS-MGA for group comparisons between Sámi and non-Sámi adolescents. Given this multi-group focus, I recommend adding a brief subsection describing how you will evaluate measurement invariance across groups before interpreting differences in structural paths. For example, you might note whether you will use the MICOM (Measurement Invariance of Composite Models) procedure or an equivalent approach to assess configural, compositional, and scalar invariance for key constructs (e.g., PIU, ethnic identity, norms). Clarifying this step will strengthen the methodological rigor of your planned group comparisons and align your protocol more closely with current recommendations in the PLS-SEM literature.*

**Response:**

Thank you for this insightful suggestion. We agree that assessing measurement invariance is critical for ensuring valid group comparisons in multigroup PLS-SEM (PLS-MGA). To address this, we have revised the manuscript to include a subsection on PLS-MGA (p.24, l.- p.25, l.11), providing a detailed description of how measurement invariance will be assessed using the Measurement Invariance of Composite Models (MICOM) procedure. The MICOM procedure involves three steps: (1) configural invariance, (2) compositional invariance, and (3) equality of composite mean values and variances. This ensures that the constructs are measured equivalently across Sámi and non-Sámi groups, allowing for meaningful interpretation of differences in structural paths. Additionally, we have outlined how multigroup analysis (MGA) will be conducted to test for significant differences in group-specific parameter estimates (e.g., outer weights, outer loadings, and path coefficients). The MGA will be performed using methods such as permutation MGA, bootstrap MGA, parametric tests, and Welch-Satterthwaite tests. These methods ensure that our group comparisons are methodologically robust and free from measurement bias.

These additions strengthen the methodological rigor of our study and align with current best practices in the PLS-SEM literature, as recommended by Henseler et al. (2016), Hair et al. (2024), and Sarstedt et al. (2011).

References

- Hair, J. F., Sarstedt, M., Ringle, C. M., & Gudergan, S. P. (2024). *Advanced Issues in Partial Least Squares Structural Equation Modeling (PLS-SEM)* (2nd ed.). Thousand Oaks, CA: Sage.
- Henseler, J., Ringle, C. M., & Sarstedt, M. (2016). Testing measurement invariance of composites using partial least squares. *International Marketing Review, 33*(3), 405–431.
- Sarstedt, M., Henseler, J., & Ringle, C. M. (2011). Multi-group analysis in partial least squares (PLS) path modeling: Alternative methods and empirical results. *Advances in International Marketing, 22*, 195–218.

**Comment 5:**

*The Data Preparation section indicates that respondents with more than 15% missing data will be excluded and that remaining missing values may be handled using multiple imputation, alongside identification and treatment of outliers and data transformations. Please specify:

(i) The operational definition of “>15% missing data” (e.g., across all items, within key scales only, or across model variables).
(ii) The planned imputation method (e.g., multiple imputation by chained equations, number of imputations, variables included in the imputation model) and whether imputation will be conducted separately within Sámi and non-Sámi groups, given that you will later compare groups.
(iii) How you will define and handle outliers (e.g., specific thresholds or influence diagnostics), particularly in the context of PLS-SEM.*

**Response:**
Thank you for this helpful request for clarification. We have revised the manuscript to define the missing data rule at the respondent level and to provide full details on our imputation plan and multigroup handling. The following details have been inserted into the Data preparation section (p. 21, l.18-p.23, l.2):

(i) Operational definition of “>15% missing data.”
Following PLS-SEM methodological guidance, which recommends removing variables or cases with >15% missingness and transparently reporting missingness at both the dataset and variable levels, we define “>15% missing data” at the respondent (record) level across all variables used in the measurement and structural models. Respondents exceeding this threshold will be excluded prior to imputation. The percentage of missing values per variable and overall will also be reported, as recommended (Ringle, Sarstedt, and Sinkovics, 2023)

(ii) Imputation method and group-specific imputation.
For remaining missing values, we will use multiple imputation by chained equations (MICE) with m = 30 imputations. This approach aligns with published recommendations that multiple imputation or maximum likelihood methods are appropriate when missingness exceeds trivial levels (i.e., beyond ~5%), and that imputation is preferred over deletion when missingness exceeds ~10% to reduce bias and preserve statistical power (Ringle, Sarstedt, and Sinkovics, 2023; Shela et al., 2023; Abreu Ledón et al., 2026).

Because our analyses compare Sámi and non-Sámi adolescents using multigroup PLS-SEM, imputation will be conducted separately within these groups. This ensures that group-specific means, variances, covariances, and distributional properties are preserved, which is essential for valid multigroup analyses and measurement invariance testing (MICOM). This approach follows PLS-SEM guidance on carefully handling grouping variables in multigroup analysis workflows (Shela et al., 2023).

(iii) Outlier identification and treatment.
We have added a concise description of our outlier handling procedures. Following PLS-SEM recommendations, we screened for suspicious response patterns (e.g., straight-lining, alternating extremes) and extreme values using descriptive statistics and visualization techniques such as boxplots, as advised in the guidance by Ringle, Sarstedt, and Sinkovics (2023).

Consistent with the review by Shela et al. (2023), which highlights that outliers can adversely affect PLS-SEM estimation and should be systematically examined, we evaluated both univariate and multivariate outliers. Only those clearly attributable to erroneous responding were removed. The review by Abreu Ledón et al. (2026) similarly emphasizes that such preprocessing is necessary to avoid biased SEM results, reinforcing our approach to outlier screening prior to model estimation.

References

- Ringle, C. M., Sarstedt, M., Sinkovics, N., & Sinkovics, R. R. (2023). A perspective on using partial least squares structural equation modelling in data articles. *Data Brief, 48*, 109074. <https://doi.org/10.1016/j.dib.2023.109074>
- Shela, V., Khatri, S., & Patel, R. (2023). Run! This road has no ending! A systematic review of PLS-SEM application in strategic management research among developing nations. *Heliyon, 9*(12), e22476. <https://doi.org/10.1016/j.heliyon.2023.e22476>
- Abreu-Ledón, R., Sánchez, M. Á. G., & Pérez, J. M. P. (2026). Evaluating the use of structural equation modeling in online purchase intention research: A comprehensive review (2000−2023). *Technological Forecasting and Social Change, 225*, 124512. <https://doi.org/10.1016/j.techfore.2026.124512>

**Minor comments
Comment 1:**

*In the submission system, the Data Availability section currently notes that no results are reported and lists “N/A.” In the protocol manuscript, it would be helpful to add a concise statement in the Methods specifying where de-identified survey data and the codebook will be deposited upon study completion (e.g., a specific institutional or national repository), in line with PLOS ONE’s open data policy.*

**Response:**
Thank you for the guidance. We have updated the manuscript to specify the data repository in accordance with PLOS ONE’s open data policy, which requires public sharing of the minimal dataset necessary to replicate the reported findings and strongly recommends deposition in a repository.

The Data availability section (p.2, l.14-18) now states that de‑identified survey data and the accompanying codebook will be deposited in the Norwegian Research Information Repository (Nasjonalt vitenarkiv, NVA)—Norway’s national open research archive operated by Sikt (the Norwegian Agency for Shared Services in Education and Research). We have also updated the Data Availability Statement in the PLOS ONE submission portal to reflect the use of this repository.

**Comment 2:**

*The Gantt chart provides a clear overview of the planned activities from piloting and questionnaire finalization (WP1) through data collection, analysis, and dissemination (WP2-WP4). You might consider briefly noting in the text that the school-based recruitment is population-based (i.e., all upper-secondary schools in Finnmark) and that the expected sample size (approximately 1,400-1,500 participants) is based on current enrollment figures, to reinforce feasibility.*

**Response:**

Thank you for this helpful suggestion. Information regarding the population‑based recruitment strategy and the rationale for the expected sample size is already presented in the Sampling Methods (p.12, l.6 – p.13, l.3) subsection of Materials and Methods. To reinforce feasibility in relation to the project timeline and the operational work packages, we have added a brief clarifying statement to the WP2: Data collection subsection (p.21, l.2-6). This added text explicitly notes that recruitment will include all high schools in Finnmark County and that the expected sample size of approximately 1,400–1,500 students is based on current enrollment figures. This clarification strengthens the alignment between the Gantt chart and the feasibility of the planned data collection.

**Comment 3:**

*The Limitations section appropriately acknowledges the cross-sectional design, self-report data, non-response bias, and the risk of type I error given multiple tests. You may wish to add a brief statement noting that, although PLS-SEM can handle complex latent structures, it does not in itself resolve the temporal ambiguity of cross-sectional data reinforcing that all modeled relationships will be interpreted as associations rather than causal effects.*

**Response:**
We thank the reviewer for this suggestion. We have added a sentence to the limitation section (p.31, l.14–16) clarifying that, although PLS-SEM can model complex latent structures, it does not resolve the temporal ambiguity of cross-sectional data; therefore, all modeled relationships are interpreted as associations rather than causal effects.

## **Reviewer 2:**

*This manuscript outlines a study protocol to investigate Problematic Internet Use (PIU) among Sámi and non-Sámi adolescents. The integration of Social Norm Theory within an Indigenous context is promising. However, the protocol requires significant clarification regarding sampling justification, construct definitions, and the distinction between perceived and objective norms.*

**Major Revisions:**
**Comment 1:**

*The title and abstract should explicitly state that this is a "Study Protocol" to ensure correct indexing and reader expectations.*

**Response**

We thank the helpful suggestion. The title and abstract have been revised to explicitly state that this manuscript is a “Study Protocol.” Specifically, “Study Protocol” has been added at the beginning of the title (p.1, l. 4), and the abstract (p. 3, l.6) has been revised to read: “This study protocol outlines a population-based cross-sectional survey investigating the associations…”. These revisions ensure accurate indexing and clearly set reader expectations.

**Comment 2:**

*The rationale for PLS-SEM should be aligned with the census approach and the expected Sámi subgroup size; currently the arguments read as disconnected.*

**Response**

Thank you for this constructive comment. We agree that the rationale for PLS‑SEM should be explicitly linked to our census (population‑based) approach and the expected size of the Sámi subgroup. We have therefore added bridging text in the Sampling methods subsection to state the census design and expected subgroup counts (p.12, l.22 – p.13, l.3), and we have inserted an explicit justification in the Statistical Approach and Sample Size Calculation subsection clarifying why PLS‑SEM (and PLS‑MGA) is appropriate given the full‑population recruitment, non‑normal survey data, and unequal group sizes (p. 13, l. 5 - 22).

Because no official statistics on ethnic identity are available at the individual level, precise estimates of the Sámi subgroup size cannot be derived, and previously stated numerical estimates have therefore been removed to avoid reliance on speculative figures. Instead, feasibility and statistical adequacy are justified based on overall school enrollment figures and the full-population design. Schools located in municipalities within the Sámi Language Act administrative area are used as contextual indicators of higher Sámi population density, without being used to estimate subgroup size directly. Based on the inverse square root method, the estimated minimum sample size of 155 refers to the minimum required sample per subgroup for stable estimation in PLS-SEM. Although the exact size of the Sámi subgroup cannot be specified, the combination of the census-based recruitment strategy, inclusion of all schools in Finnmark, and prior experience from pretest and pilot phases supports the feasibility of achieving a sufficient subgroup size for the planned analyses. This explanation has been incorporated into the *Sampling Methods* subsection (p. 12, l.13–21).

**Comment 3:**

*The study aims to measure norms across family, peer, and school contexts. It is important to clarify that the study is measuring perceived norms (the adolescent's perception of others' behaviors/attitudes) rather than objective norms (gathered from teachers or parents directly).*

**Response:**
Thank you for this important comment. We have clarified that the study measures perceived norms—adolescents’ perceptions of others’ behaviors and attitudes—across family, peer, and school contexts, rather than objective norms collected directly from parents, peers, or teachers. This clarification has been added at the beginning of the Social Norms subsection of Measuring instrument in Methods (p. 19, l. 8-11) and incorporated into the second limitation in the Discussion section to highlight that perceived and objective norms may diverge (p.30, l.13-18).

**Comment 4:**

*The Introduction lists PIU alongside behaviors such as gaming, gambling, pornography use, and cyberbullying. Please clarify how you are defining PIU in this protocol and ensure the examples align with the construct measured by the PIUQ (i.e., whether these are meant as components of PIU or simply related online behaviors/outcomes).*

**Response**

Thank you for this helpful comment. We have clarified that, in this protocol, Problematic Internet Use (PIU) is defined conceptually as a pattern of excessive, compulsive, and difficult‑to‑control online behavior associated with psychosocial and functional problems (Introduction, p.4, l.7-19). Gaming, gambling, pornography consumption, and cyberbullying are presented as illustrative online contexts and related outcomes in which PIU may manifest, not as diagnostic components of the construct. In the Problematic internet use (PIU) subsection in Methods, we specify that generalized PIU will be operationalized with the PIUQ-9 and briefly describe its three symptom dimensions (obsession, neglect, control disorder**) (p.17, l.18 – p.18, l.1).**

**Comment 5:**

*The authors include "managing emotions" as an indicator of positive internet use. Please provide theoretical support for this inclusion. In clinical literature, using the internet for emotional regulation is often considered a maladaptive coping mechanism associated with PIU, rather than a protective factor.*

**Author response:**
Thank you for this clarification request. In our study, “managing emotions” is not a standalone indicator. The construct we measure is positive internet use, defined as adaptive and supportive digital engagement (e.g., maintaining social connections, seeking social support, accessing culturally meaningful content, identity expression, and learning). Any reference to “managing emotions” is illustrative of supportive contexts (e.g., receiving help when stressed or lonely) and is conceptually distinct from avoidance‑based or compulsive emotion regulation associated with PIU.

To address this concern and prevent ambiguity, we have made the following revisions:

1. Reworded H3: The phrase “manage emotions” has been replaced with “seek social support and emotional connection” to better reflect the construct’s intended meaning (p.11, l.18–20).
2. Clarified in the Measuring Instrument Section: We have added a subsection on Positive Internet Use, operationalizing it as a separate latent construct in the structural models. This construct includes maintaining social connections, seeking social support and emotional connection, and engaging with cultural identity (p.18, l.19–p.19, l.6).

**Comment 6:**

*The subjective self-identification criterion classifies adolescents as Sámi only if they endorse "Fully agree" or "Nearly fully agree" items. Please provide a citation or stronger theoretical justification for excluding participants who might answer "Somewhat agree" as this risks excluding adolescents with exploring or dual identities.*

**Response**

Thank you for this thoughtful comment. We recognize that classifying Sámi identity based solely on high levels of endorsement (e.g., “fully” or “nearly fully agree”) risks excluding adolescents who are in the process of identity exploration or who hold plural (bicultural/multiethnic) identities. Both identity exploration and plural identities are normative during adolescence, as highlighted in developmental models of ethnic identity (e.g., Phinney, 1993). According to Phinney’s model, exploration and commitment are distinct processes, and adolescents may authentically identify with an ethnic group while still exploring their identity. Imposing a high endorsement threshold may therefore result in misclassification.

To address this limitation, we have revised the measurement of self-identification by replacing the graded agreement item with a multiple-choice ethnicity question, allowing participants to select one or more categories (e.g., Sámi, Norwegian, Kven, etc.). This approach better captures the complexity and fluidity of ethnic identity, avoids arbitrary thresholds, and aligns with best practices in ethnicity research.

This revision is reflected in the Classification of Sámi and non-Sámi adolescents in WP1.1 Measuring instruments (p.16-17) and specifically in the updated description of the ethnicity measure (p.17, l.1–14).

**Reference:**

- Phinney, J. S. (1993). A three-stage model of ethnic identity development in adolescence. In M. E. Bernal & G. P. Knight (Eds.), *Ethnic identity: Formation and transmission among Hispanics and other minorities* (pp. 61–79). Albany, NY: SUNY Press.

**Comment 7:**

*One of the primary aims of the study is to assess the prevalence of PIU. However, the protocol does not specify the cut-off scores or diagnostic criteria that will be used to classify participants as having "problematic" versus "non-problematic" use based on the PIUQ. Establishing and pre-registering these thresholds based on existing literature is essential for the validity of prevalence estimates.*

**Response:**

Thank you for your comment. We agree that establishing and pre-registering thresholds is important for assessing the prevalence of Problematic Internet Use (PIU). Below, we outline our approach:

1. Cut-Off Score for PIUQ-9:
   For descriptive purposes, we will use a pre-registered cut-off score of ≥ 22 on the PIUQ-9 (range: 9–45) to classify participants as being "at risk" of problematic internet use. This threshold is based on proportional scaling from the established cut-off for the PIUQ-18 (≥ 41; Koronczai et al., 2011). While the PIUQ-9 has demonstrated robust psychometric properties, no universally accepted diagnostic cut-off exists (Laconi et al., 2019; Burkauskas et al., 2020). Therefore, this cut-off will serve as a descriptive benchmark, not a diagnostic criterion.
2. Continuous Treatment in Models:
   In all structural models, PIU will be treated as a continuous latent variable to capture the full range of variability in participants' responses, aligning with the psychometric properties of the PIUQ-9.

This approach ensures both the validity of prevalence estimates and the flexibility to analyze PIU as a continuous construct in structural models.

These responses have been reflected into the measurement of PIU (p.18, l.4-13) in WP1.1: Measuring instruments, Descriptive statistics (p.23, l.3–10) and PLS-SEM (p.23, l.12-14) in Data analysis.

References

- Koronczai, B., et al. (2011). Confirmation of the three-factor model of problematic internet use on offline adolescent and adult samples. *Cyberpsychology, Behavior, and Social Networking, 14*(11), 657–664.
- Laconi, S., et al. (2019). Psychometric evaluation of the nine-item Problematic Internet Use Questionnaire (PIUQ-9) in nine European samples of internet users. *Frontiers in Psychiatry, 10*, 136.
- Burkauskas, J., et al. (2020). Problematic internet use and its relationship with health-related quality of life in a cross-sectional sample: Mediating role of behavioral avoidance. *International Journal of Environmental Research and Public Health, 17*(22), 8617.

**Comment 8:**

*The conceptual framework (Fig. 1) and the analysis plan connect norms, identity, and victimization directly to PIU without mentioning adjustment for potential confounding variables (gender, socioeconomic status, anxiety/depression, etc.). The authors should explicitly state which control variables will be included in the structural model or provide a rigorous justification for their exclusion.*

**Response**

Thank you for this important comment. We agree that potential confounding should be addressed explicitly. In line with prior work on digital and social media use among adolescents in Finnmark, our structural models will adjust for the following individual covariates: gender, grade level (VG1–VG3), living arrangement (with parents vs. dormitory), depressive thoughts, alcohol use, and smoking/nicotine use. Items for these covariates will be drawn from Ungdata, a widely used national monitoring system for adolescent health and well‑being (permission obtained).

In addition, we have clarified and expanded the inclusion of contextual-level factors. Specifically, *ethnic community context* is included as a school-level control variable, operationalized based on whether the school is located in a municipality within the Sámi Language Act administrative area. This classification is used as a proxy for higher Sámi population density (i.e., Sámi-dominated vs. more Norwegian-dominated areas). Schools located in Kautokeino, Karasjok, Lakselv (Porsanger), and Tana are therefore categorized as higher Sámi density, while schools in other municipalities are categorized as lower Sámi density. This variable is included to account for geographic and sociocultural variation in adolescents’ environments without expanding the number of analytical groups.

Accordingly, all control variables—both individual and contextual—are entered as exogenous covariates with direct paths to PIU in the primary PLS-SEM specification, while ethnic community context is not used for subgroup or multigroup analyses due to sample size considerations.

We have added these details to Control variables section in Measuring instruments (p. 20, l.8-25) and to PLS-SEM section in Data analysis (p. 25, l. 9-11).

**Minor Revisions:
Comment 1:**

*Avoid using "(personal communication, 2025)" in the Introduction to establish the research problem. Please rely on pilot data or existing literature.*

**Response**

Thank you for this helpful comment. The sentences citing “personal communication, 2025” have been removed from the section **Internet Use in Finnmark** in Introduction.

Since this description primarily relates to the positive aspects of internet use rather than PIU, and given the limited availability of pilot data or published literature addressing both positive and negative aspects of digital media use within the Sámi context, this discussion has been relocated to the subsequent section, Internet Use and Indigenous Adolescents (p. 6, l.13-19). This ensures that the discussion is framed appropriately and supported by existing literature where possible.

**Comment 2:**

*The terms "VG1–VG3" are specific to the Norwegian education system. Please provide the international equivalent for a global audience.*

**Response**

Thank you for highlighting this important point. To enhance clarity for an international audience, we have added the equivalent terms “11th–13th grade” following “VG1–VG3” in the Sampling Methods section (p. 6, l.7) and Control variables section (p. 20, l.11).

**Comment 3:**

*The statement regarding "excessive reliance on technology…" (Introduction) should be nuanced with more recent, critical literature that avoids technological determinism.*

**Response**

Thank you for your valuable comment. I have revised the statement to avoid technological determinism by framing it more critically and in line with recent literature. For example, it now reads: *“PIU is further linked to increased engagement in risky behaviors (e.g., cyberbullying and desensitization to aggression through violent media exposure) [9], sleep disturbances [10], and impairments in social and school functioning [6], including reduced face to face interaction [11].”* (p. 4, l.13-16)

**Comment 4:**

*Given the significant effort described in Work Package 1 to develop a culturally adapted questionnaire in both Norwegian and Northern Sámi, it would be valuable if the authors could explicitly state whether this instrument will be made open access upon publication of the results.*

**Response:**
We thank the reviewer for highlighting the importance of accessibility of the culturally adapted questionnaire. We have added a statement in the Methods section, under WP1: Instrument Development, to clarify that the instrument will be openly available (p.16, l.7–8).

**Comment 5:**

*The protocol mentions conducting a pretest and a pilot test with approximately 30 students each (WP1). Please explicitly state whether these students will be excluded from the main study sample (WP2). If they are to be included, please justify this decision and discuss potential limitations.*

**Response**

Thank you for this important comment. The pretest and pilot test are conducted during the 2025–2026 school year and primarily involve students in the highest grade level (VG3). As the main study (WP2) will take place in 2027, these students will have graduated before the WP2 data collection begins and will therefore not be included in the main study sample. This clarification has been added to the Methods section under WP1: Instrument Development, at the end of the paragraph describing the pretest and pilot test (p.16, l.3-6).

**Comment 6:**

*The section on data cleaning is generic. Please specify the criteria for excluding outliers and the specific method of multiple imputation intended.*

**Response:**
Thank you for this helpful request for clarification. We recognize the importance of providing detailed and transparent descriptions of data cleaning procedures, including the criteria for excluding outliers and the specific method of multiple imputation. In response to this comment, as well as similar feedback received from another reviewer, we have revised the manuscript to define the missing data rule at the respondent level and to provide full details on our imputation plan and outlier handling procedures. These revisions have been incorporated into the Data preparation section (p. 21, l.18-p.23, l.2):

(i) Operational definition of “>15% missing data.”
Following PLS-SEM methodological guidance, which recommends removing variables or cases with >15% missingness and transparently reporting missingness at both the dataset and variable levels, we define “>15% missing data” at the respondent (record) level across all variables used in the measurement and structural models. Respondents exceeding this threshold will be excluded prior to imputation. The percentage of missing values per variable and overall will also be reported, as recommended (Ringle, Sarstedt, and Sinkovics, 2023)

(ii) Imputation method and group-specific imputation.
For remaining missing values, we will use multiple imputation by chained equations (MICE) with m = 30 imputations. This approach aligns with published recommendations that multiple imputation or maximum likelihood methods are appropriate when missingness exceeds trivial levels (i.e., beyond ~5%), and that imputation is preferred over deletion when missingness exceeds ~10% to reduce bias and preserve statistical power (Ringle, Sarstedt, and Sinkovics, 2023; Shela et al., 2023; Abreu Ledón et al., 2026).

Because our analyses compare Sámi and non-Sámi adolescents using multigroup PLS-SEM, imputation will be conducted separately within these groups. This ensures that group-specific means, variances, covariances, and distributional properties are preserved, which is essential for valid multigroup analyses and measurement invariance testing (MICOM). This approach follows PLS-SEM guidance on carefully handling grouping variables in multigroup analysis workflows (Shela et al., 2023).

(iii) Outlier identification and treatment.
We have added a concise description of our outlier handling procedures. Following PLS-SEM recommendations, we screened for suspicious response patterns (e.g., straight-lining, alternating extremes) and extreme values using descriptive statistics and visualization techniques such as boxplots, as advised in the guidance by Ringle, Sarstedt, and Sinkovics (2023).

Consistent with the review by Shela et al. (2023), which highlights that outliers can adversely affect PLS-SEM estimation and should be systematically examined, we evaluated both univariate and multivariate outliers. Only those clearly attributable to erroneous responding were removed. The review by Abreu Ledón et al. (2026) similarly emphasizes that such preprocessing is necessary to avoid biased SEM results, reinforcing our approach to outlier screening prior to model estimation.

References

- Ringle, C. M., Sarstedt, M., Sinkovics, N., & Sinkovics, R. R. (2023). A perspective on using partial least squares structural equation modelling in data articles. *Data Brief, 48*, 109074. <https://doi.org/10.1016/j.dib.2023.109074>
- Shela, V., Khatri, S., & Patel, R. (2023). Run! This road has no ending! A systematic review of PLS-SEM application in strategic management research among developing nations. *Heliyon, 9*(12), e22476. <https://doi.org/10.1016/j.heliyon.2023.e22476>
- Abreu-Ledón, R., Sánchez, M. Á. G., & Pérez, J. M. P. (2026). Evaluating the use of structural equation modeling in online purchase intention research: A comprehensive review (2000−2023). *Technological Forecasting and Social Change, 225*, 124512. <https://doi.org/10.1016/j.techfore.2026.124512>

**Comment 7:**

*There is a discrepancy between the text and the Gantt Chart (Table 1). The manuscript states that "Cultural Adaptation, Translation, and Pretesting" are "to be completed in 2025" (WP1 description). However, Table 1 schedules WP1 to run throughout almost the entire year of 2026. Additionally, given the current date, the timeline should be reviewed to ensuring it remains prospective and realistic for the project's actual start date.*

**Response**

Thank you for pointing this out. We have corrected the discrepancy between the text and the Gantt chart. In addition, the project timeline has been slightly revised to ensure consistency and feasibility. These updates have been reflected in the Gantt chart (p.26) as well as in the corresponding sections of the manuscript, including Cultural Adaptation, Translation, and Pretesting (p.15, l.3), which is now scheduled to be completed in the first quarter of 2026, and “Data Collection” (p.21, l.5-6).

**Comment 8:**

*Please correct punctuation errors, such as the double period on page 7 ("values [46, 47]..") and the semicolon usage on page 4.*

**Response**

Thank you for pointing this out. The punctuation error, specifically the double period on page 8, “values [46, 47]..,” has been corrected to “values [44, 45]” (the original citation numbers [46, 47] have been updated to [44, 45] in the revised version) (p.8, l.5).

Additionally, the semicolon on page 5 (p.5, l.2) has been replaced with a full stop to improve clarity and flow. The revised text now reads as two separate sentences: “Schools also moderate PIU through supportive environments [16]. For example, shared gaming interests within classrooms may foster social connection and reduce PIU symptoms [17].”

# **Other revisions:**

**Clarification on School Absence, Dropout, and Ethnic Identity Disclosure**

In the third limitation paragraph (p. 30, l. 22-p.31, l.5), we have clarified that adolescents who are absent on the day of data collection (e.g., due to truancy, irregular attendance, or sleep-related problems) or who have already dropped out of high school are not included in the sample. We also note that these groups may have higher levels of problematic internet use.

**Abstract Revision**

The Abstract has been revised to reflect the updates and changes made throughout the manuscript.
