# Supplementary material for: Risk and resilience factors in problematic internet use among Sámi and non-Sámi adolescents in Finnmark, Arctic Norway: The role of social norms and ethnic identity
Source: PLoS One. 2026 Aug 26;21(8):e0356920. doi: 10.1371/journal.pone.0356920 (PMC13513970; doi:10.1371/journal.pone.0356920)
Supplement: Supplementary file 3 [file pone.0356920.s003.docx]

**Response to the Reviewers’ Comments**

## **Reviewer 1**:

*Thank you for the thorough and constructive revision. The manuscript is much improved, and my substantive concerns have been addressed.
I have no further substantive comments. Before publication, please remove or resolve the residual phrase “rural (?)” in the Discussion. You may also consider adding one brief sentence noting whether measurement invariance across questionnaire language versions will be explored if sample size permits, or otherwise acknowledged as a limitation.*

**Response**

Thank you for your positive assessment of the revised manuscript and for your helpful suggestions. The residual phrase “rural (?)” in the Discussion has been corrected and now reads: “The study is relevant for both regional and broader efforts to promote digital well-being among adolescents in Arctic, rural, and Indigenous contexts.” (p. 30, l. 4-5)

Regarding measurement invariance across questionnaire language versions, we have added a statement to the limitations of the Discussion (p.30, l.23 – p.31, l.2). Although the questionnaire has undergone forward–backward translation and cultural adaptation, the number of participants completing the Northern Sámi version may be limited. Consequently, formal assessment of measurement invariance across language versions may not be feasible for all constructs.

## **Reviewer 2**:

*I thank the authors for the substantial effort invested in this revision. The manuscript is clearer and several of my previous concerns have been adequately addressed, particularly the distinction between perceived and objective norms, the definition of PIU, and the revised approach to Sámi self-identification.
A few issues still require clarification before the protocol is fully ready. First, the 'positive internet use' construct still needs a clearer theoretical and measurement rationale, including the full items and a pre-specified validation strategy; an EFA in a very small pilot sample would not be sufficient for this purpose.
Second, the proposed cut-off score (≥22) for the PIUQ-9, derived via proportional scaling from the PIUQ-18, lacks psychometric robustness. Even if used strictly for descriptive purposes, this threshold remains methodologically fragile. The authors should either provide a stronger empirical justification for this specific cut-off or explicitly acknowledge this limitation regarding the validity of their prevalence estimates.*

**Response:**
Thank you for your constructive feedback and for recognizing the substantial revisions made to the manuscript.

We agree that the rationale and validation strategy for the positive internet use construct required further clarification. We have revised the manuscript to describe positive internet use as a conceptually and empirically informed construct derived from previous research on Indigenous youth, rather than as an established psychometric scale. This clarification has been incorporated into the WP1.1: Measuring Instruments section of the Methods (p.18, l.19 – p.19, l.11). Previous studies among Indigenous youth in Australia and Canada have shown that digital technologies may promote social connection, emotional support, community belonging, cultural engagement, and identity affirmation (Rice et al., 2016; Castleton, 2018; Johnson, 2016; Paul et al., 2024). Based on this literature, positive internet use is conceptualized as adolescents' use of digital technologies to maintain social connections, seek social and emotional support, and engage with cultural identity. These domains were further refined through interviews with Sámi and non-Sámi adolescents to ensure cultural relevance in the Finnmark context.

We have also revised the validation strategy. Rather than relying on exploratory factor analysis (EFA) in the small pilot sample, the pilot phase will be used primarily to assess item clarity, relevance, and feasibility. The dimensionality, reliability, and construct validity of the positive internet use items will subsequently be evaluated in the full study sample prior to inclusion in the structural models. In line with the study protocol's commitment to transparency, the Norwegian and Northern Sámi versions of the questionnaire will be made openly accessible for research purposes following completion of the study and publication of the results.

Regarding the PIUQ-9 cut-off score, we agree that the proposed threshold (≥22), derived through proportional scaling from the PIUQ-18, has limited psychometric validation. We have therefore revised the manuscript to acknowledge this limitation more explicitly. The manuscript now clarifies that no universally validated cut-off currently exists for the PIUQ-9 and that the threshold of ≥22 will be used solely as a descriptive benchmark rather than as a diagnostic criterion. We have further noted in the limitations in the Discussion section that prevalence estimates based on this threshold should be interpreted with caution due to the limited empirical validation of the cut-off (p.31, l.14-16).

**References cited in this response**

- Castleton, A. (2018). *Technology and Inuit identity: Facebook use by Inuit youth*. AlterNative, 14(3), 228–236.
- Johnson, G. M. (2016). *Technology use among Indigenous adolescents in remote regions of Australia*. International Journal of Adolescence and Youth, 21(2), 218–231.
- Paul, J., McQuaid, R. J., Hopkins, C., Perri, A., Stewart, S., Matheson, K., Anisman, H., & Bombay, A. (2024). *Relations between bullying and distress among youth living in First Nations communities: Assessing direct and moderating effects of culture-related variables*. Transcultural Psychiatry, 61(3), 429–439.
- Rice, E. S., Haynes, E., Royce, P., & Thompson, S. C. (2016). *Social media and digital technology use among Indigenous young people in Australia: A literature review*. International Journal for Equity in Health, 15, 81.
