# Supplementary material for: Risk and resilience factors in problematic internet use among Sámi and non-Sámi adolescents in Finnmark, Arctic Norway: The role of social norms and ethnic identity
Source: PLoS One. 2026 Aug 26;21(8):e0356920. doi: 10.1371/journal.pone.0356920 (PMC13513970; doi:10.1371/journal.pone.0356920)
Supplement: Peer review history

# Author comment: Author response to Decision Letter 0

**DOI:** 10.1371/journal.pone.0356920.r001

27 Sep 2025

Attachment Submitted filename: Author Response to Requested Revisions .pdf

# Peer review: Decision Letter 0

**DOI:** 10.1371/journal.pone.0356920.r002

**Authors:** José Perales

8 Jan 2026

Dear Dr. Hansen,

plosone@plos.org. When you're ready to submit your revision, log on to https://www.editorialmanager.com/pone/ and select the 'Submissions Needing Revision' folder to locate your manuscript file.

- A letter that responds to each point raised by the academic editor and reviewer(s). You should upload this letter as a separate file labeled 'Response to Reviewers'.
- A marked-up copy of your manuscript that highlights changes made to the original version. You should upload this as a separate file labeled 'Revised Manuscript with Track Changes'.
- An unmarked version of your revised paper without tracked changes. You should upload this as a separate file labeled 'Manuscript'.

If you would like to make changes to your financial disclosure, please include your updated statement in your cover letter. Guidelines for resubmitting your figure files are available below the reviewer comments at the end of this letter.

If applicable, we recommend that you deposit your laboratory protocols in protocols.io to enhance the reproducibility of your results. Protocols.io assigns your protocol its own identifier (DOI) so that it can be cited independently in the future. For instructions see: https://journals.plos.org/plosone/s/submission-guidelines#loc-laboratory-protocols. Additionally, PLOS ONE offers an option for publishing peer-reviewed Lab Protocol articles, which describe protocols hosted on protocols.io. Read more information on sharing protocols at https://plos.org/protocols?utm_medium=editorial-email&utm_source=authorletters&utm_campaign=protocols.

We look forward to receiving your revised manuscript.

Kind regards,

José C. Perales

Academic Editor

PLOS One

Journal requirements:

When submitting your revision, we need you to address these additional requirements.

1. Please ensure that your manuscript meets PLOS ONE's style requirements, including those for file naming. The PLOS ONE style templates can be found at

https://journals.plos.org/plosone/s/file?id=wjVg/PLOSOne_formatting_sample_main_body.pdf and

https://journals.plos.org/plosone/s/file?id=ba62/PLOSOne_formatting_sample_title_authors_affiliations.pdf

2. Kindly amend the title of your manuscript to include the word "protocol" in it.

3. Thank you for stating the following in the Acknowledgments Section of your manuscript:

“We gratefully acknowledge the support of the Research and Development Fund of the Sámi Norwegian National Advisory Unit for Mental Health and Substance Use (SANKS), which enabled the preparation of this research protocol. We also thank the young people who participated in the participatory meetings for their valuable contributions to the development of the questionnaire. We further acknowledge Ronald Isaksen, Administrative Coordinator at SANKS, for his role in organizing and facilitating meetings with youth and schools. Finally, we appreciate the Research Department at Finnmark Hospital Trust for providing a supportive and enabling research environment.”

We note that you have provided funding information that is currently declared in your Funding Statement. However, funding information should not appear in the Acknowledgments section or other areas of your manuscript. We will only publish funding information present in the Funding Statement section of the online submission form.

Please remove any funding-related text from the manuscript and let us know how you would like to update your Funding Statement. Currently, your Funding Statement reads as follows:

“The development of the study protocol was supported by the Research and Development Fund of the Sámi Norwegian National Advisory Unit for Mental Health and Substance Use (SANKS), Finnmark Hospital Trust. The funders had no role in the analysis or interpretation of data, nor did they have any authority over the writing or publication of the manuscript. No additional external funding was received for this study. Funding for the study itself will primarily be provided by Helse Nord, supplemented by institutional research funds from SANKS and Finnmark Hospital Trust. Publication fees and indirect costs will be covered by the hosting institutions.”

Please include your amended statements within your cover letter; we will change the online submission form on your behalf.

4. If the reviewer comments include a recommendation to cite specific previously published works, please review and evaluate these publications to determine whether they are relevant and should be cited. There is no requirement to cite these works unless the editor has indicated otherwise.

[Note: HTML markup is below. Please do not edit.]

Reviewers' comments:

Reviewer's Responses to Questions

**Comments to the Author**

1. Does the manuscript provide a valid rationale for the proposed study, with clearly identified and justified research questions?

Reviewer #1: Yes

Reviewer #2: Yes

**********

2. Is the protocol technically sound and planned in a manner that will lead to a meaningful outcome and allow testing the stated hypotheses?

Reviewer #1: Yes

Reviewer #2: Partly

**********

3. Is the methodology feasible and described in sufficient detail to allow the work to be replicable?

Reviewer #1: Yes

Reviewer #2: No

**********

4. Have the authors described where all data underlying the findings will be made available when the study is complete?

The PLOS Data policy

Reviewer #1: Yes

Reviewer #2: No

**********

5. Is the manuscript presented in an intelligible fashion and written in standard English?

Reviewer #1: Yes

Reviewer #2: Yes

**********

Please use the space provided to explain your answers to the questions above and, if applicable, provide comments about issues authors must address before this protocol can be accepted for publication. You may also include additional comments for the author, including concerns about research or publication ethics.

You may also provide optional suggestions and comments to authors that they might find helpful in planning their study.

Reviewer #1: This is a clearly written and thoughtfully designed protocol focusing on PIU and positive internet use among Sámi and non-Sámi adolescents in Finnmark. The integration of social norms theory and ethnic identity constructs is well articulated, and the cultural and ethical considerations for Indigenous research are carefully addressed.

Below are several major and minor comments that I believe will strengthen the manuscript and improve its clarity and reproducibility.

Major comments

1. Clarify the operationalization of PIU and positive internet use. You state that PIU will be measured using the Problematic Internet Use Questionnaire, Youth (PIUQ-Youth), and that positive internet use will be captured via items reflecting constructive, identity-supporting, and socially connective internet activities. Please specify explicitly:

(i) Whether the primary PIU outcome in the PLS-SEM models will be a latent factor, a total scale score, or both.

(ii) Whether you plan to use any clinical or empirical cut-off to define “problematic” vs. “non-problematic” internet use (even if only for descriptive purposes), or if PIU will be treated purely as a continuous construct.

(iii) How you will operationalize positive internet use in the structural models (e.g., separate latent factor, composite score, or multiple observed indicators).

Adding these details will make the analytic plan more transparent and help readers interpret effect sizes and prevalence estimates.

2. In your Introduction, you correctly note that PIU is associated with mental health difficulties and some health-risk behaviors, and you reference prior work on PIU in adolescents and young adults. To further strengthen this section, I recommend adding a large epidemiologic study that examined both PIU-substance misuse associations and race/ethnicity-specific prevalence, including Indigenous college students:

Qeadan F, Egbert J, English K. (2022). “Associations between problematic internet use and substance misuse among US college students.” Computers in Human Behavior, 134, 107327.

This study uses data from over 400,000 U.S. college students to quantify the associations between PIU and multiple forms of substance misuse and substance use disorder, and report PIU prevalence across demographic subgroups, including a specific race/ethnicity category for Indigenous students (“NH AI/AN/NH” in Table 1). Including this reference would not only situate your work within the broader PIU-substance use literature for youth and young adults, but also provide readers with complementary prevalence estimates of PIU among Indigenous college students in another national context, which aligns closely with your focus on Sámi adolescents.

3. The protocol defines Sámi identity based on both objective (ethnic background, language use, ancestry) and subjective (self-identification) criteria, and classifies participants as Sámi if they meet either objective or subjective criteria (or both). Non-Sámi participants are those who report no Sámi ethnic affiliation, language ties, or self-identification as Sámi. Given that your primary analyses will compare Sámi and non-Sámi groups via PLS-MGA, please clarify whether you will:

(i) Distinguish participants who meet only objective criteria from those who meet only subjective criteria, at least in descriptive statistics; and

(ii) Consider sensitivity analyses (e.g., restricting the Sámi group to those meeting both objective and subjective criteria) to assess robustness of any group differences.

Even if your primary analysis retains a single Sámi category, specifying these planned descriptive and sensitivity analyses would help readers appreciate the complexity of Indigenous identity and the potential heterogeneity within the Sámi group.

4. You plan to use PLS-SEM to model the relationships among social norms, ethnic identity, ethnic victimization, and PIU/positive internet use, and to employ PLS-MGA for group comparisons between Sámi and non-Sámi adolescents. Given this multi-group focus, I recommend adding a brief subsection describing how you will evaluate measurement invariance across groups (and languages) before interpreting differences in structural paths. For example, you might note whether you will use the MICOM (Measurement Invariance of Composite Models) procedure or an equivalent approach to assess configural, compositional, and scalar invariance for key constructs (e.g., PIU, ethnic identity, norms). Clarifying this step will strengthen the methodological rigor of your planned group comparisons and align your protocol more closely with current recommendations in the PLS-SEM literature.

5. The Data Preparation section indicates that respondents with more than 15% missing data will be excluded and that remaining missing values may be handled using multiple imputation, alongside identification and treatment of outliers and data transformations. Please specify:

(i) The operational definition of “>15% missing data” (e.g., across all items, within key scales only, or across model variables).

(ii) The planned imputation method (e.g., multiple imputation by chained equations, number of imputations, variables included in the imputation model) and whether imputation will be conducted separately within Sámi and non-Sámi groups, given that you will later compare groups.

(iii) How you will define and handle outliers (e.g., specific thresholds or influence diagnostics), particularly in the context of PLS-SEM.

Minor comments

1. In the submission system, the Data Availability section currently notes that no results are reported and lists “N/A.” In the protocol manuscript, it would be helpful to add a concise statement in the Methods specifying where de-identified survey data and the codebook will be deposited upon study completion (e.g., a specific institutional or national repository), in line with PLOS ONE’s open data policy.

2. The Gantt chart provides a clear overview of the planned activities from piloting and questionnaire finalization (WP1) through data collection, analysis, and dissemination (WP2-WP4). You might consider briefly noting in the text that the school-based recruitment is population-based (i.e., all upper-secondary schools in Finnmark) and that the expected sample size (approximately 1,400-1,500 participants, with 360-390 Sámi) is based on current enrollment figures, to reinforce feasibility.

3. The Limitations section appropriately acknowledges the cross-sectional design, self-report data, non-response bias, and the risk of type I error given multiple tests. You may wish to add a brief statement noting that, although PLS-SEM can handle complex latent structures, it does not in itself resolve the temporal ambiguity of cross-sectional data reinforcing that all modeled relationships will be interpreted as associations rather than causal effects.

Reviewer #2: This manuscript outlines a study protocol to investigate Problematic Internet Use (PIU) among Sámi and non-Sámi adolescents. The integration of Social Norm Theory within an Indigenous context is promising. However, the protocol requires significant clarification regarding sampling justification, construct definitions, and the distinction between perceived and objective norms.

Major Revisions:

1. The title and abstract should explicitly state that this is a "Study Protocol" to ensure correct indexing and reader expectations.

2. The rationale for PLS-SEM should be aligned with the census approach and the expected Sámi subgroup size; currently the arguments read as disconnected.

3. The study aims to measure norms across family, peer, and school contexts. It is important to clarify that the study is measuring perceived norms (the adolescent's perception of others' behaviors/attitudes) rather than objective norms (gathered from teachers or parents directly).

4. The Introduction lists PIU alongside behaviors such as gaming, gambling, pornography use, and cyberbullying. Please clarify how you are defining PIU in this protocol and ensure the examples align with the construct measured by the PIUQY (i.e., whether these are meant as components of PIU or simply related online behaviors/outcomes).

5. The authors include "managing emotions" as an indicator of positive internet use. Please provide theoretical support for this inclusion. In clinical literature, using the internet for emotional regulation is often considered a maladaptive coping mechanism associated with PIU, rather than a protective factor.

6. The subjective self-identification criterion classifies adolescents as Sámi only if they endorse "Fully agree" or "Nearly fully agree" items. Please provide a citation or stronger theoretical justification for excluding participants who might answer "Somewhat agree" as this risks excluding adolescents with exploring or dual identities.

7. One of the primary aims of the study is to assess the prevalence of PIU. However, the protocol does not specify the cut-off scores or diagnostic criteria that will be used to classify participants as having "problematic" versus "non-problematic" use based on the PIUQY. Establishing and pre-registering these thresholds based on existing literature is essential for the validity of prevalence estimates.

8. The conceptual framework (Fig. 1) and the analysis plan connect norms, identity, and victimization directly to PIU without mentioning adjustment for potential confounding variables (gender, socioeconomic status, anxiety/depression, etc.). The authors should explicitly state which control variables will be included in the structural model or provide a rigorous justification for their exclusion.

Minor Revisions:

1. Avoid using "(personal communication, 2025)" in the Introduction to establish the research problem. Please rely on pilot data or existing literature.

2. The terms "VG1–VG3" are specific to the Norwegian education system. Please provide the international equivalent for a global audience.

3. The statement regarding "excessive reliance on technology…" (Introduction) should be nuanced with more recent, critical literature that avoids technological determinism.

4. Given the significant effort described in Work Package 1 to develop a culturally adapted questionnaire in both Norwegian and Northern Sámi, it would be valuable if the authors could explicitly state whether this instrument will be made open access upon publication of the results.

5. The protocol mentions conducting a pretest and a pilot test with approximately 30 students each (WP1). Please explicitly state whether these students will be excluded from the main study sample (WP2). If they are to be included, please justify this decision and discuss potential limitations.

6. The section on data cleaning is generic. Please specify the criteria for excluding outliers and the specific method of multiple imputation intended.

7. There is a discrepancy between the text and the Gantt Chart (Table 1). The manuscript states that "Cultural Adaptation, Translation, and Pretesting" are "to be completed in 2025" (WP1 description). However, Table 1 schedules WP1 to run throughout almost the entire year of 2026. Additionally, given the current date, the timeline should be reviewed to ensuring it remains prospective and realistic for the project's actual start date.

8. Please correct punctuation errors, such as the double period on page 7 ("values [46, 47]..") and the semicolon usage on page 4.

I wish the research team the best of luck with the upcoming phases of this project. I am available to review a revised version of this manuscript if the editors consider it appropriate.

**********

what does this mean?). If published, this will include your full peer review and any attached files.

If you choose “no”, your identity will remain anonymous but your review may still be made public.

**Do you want your identity to be public for this peer review?**  For information about this choice, including consent withdrawal, please see our Privacy Policy

Reviewer #1: No

Reviewer #2: **Yes:**  Jose López-Guerrero

**********

[NOTE: If reviewer comments were submitted as an attachment file, they will be attached to this email and accessible via the submission site. Please log into your account, locate the manuscript record, and check for the action link "View Attachments". If this link does not appear, there are no attachment files.]

To ensure your figures meet our technical requirements, please review our figure guidelines: https://journals.plos.org/plosone/s/figures

You may also use PLOS’s free figure tool, NAAS, to help you prepare publication quality figures: https://journals.plos.org/plosone/s/figures#loc-tools-for-figure-preparation.

NAAS will assess whether your figures meet our technical requirements by comparing each figure against our figure specifications.

# Author comment: Author response to Decision Letter 1

**DOI:** 10.1371/journal.pone.0356920.r003

8 Jun 2026

Editor’s comments

Comment 1:

Journal requirements:

When submitting your revision, we need you to address these additional requirements.

1. Please ensure that your manuscript meets PLOS ONE's style requirements, including those for file naming. The PLOS ONE style templates can be found at

https://journals.plos.org/plosone/s/file?id=wjVg/PLOSOne_formatting_sample_main_body.pdf and

https://journals.plos.org/plosone/s/file?id=ba62/PLOSOne_formatting_sample_title_authors_affiliations.pdf

Response:

We thank the editorial office for the additional guidance. The manuscript has been fully revised to comply with PLOS ONE formatting and style guidelines, including use of the official templates and correct file naming conventions.

Comment 2:

Kindly amend the title of your manuscript to include the word "protocol" in it.

Response:

We thank the editorial office for the helpful suggestion. “Study Protocol” has been added at the beginning of the title (p. 1, l. 4).

Comment 3:

Thank you for stating the following in the Acknowledgments Section of your manuscript:

“We gratefully acknowledge the support of the Research and Development Fund of the Sámi Norwegian National Advisory Unit for Mental Health and Substance Use (SANKS), which enabled the preparation of this research protocol. We also thank the young people who participated in the participatory meetings for their valuable contributions to the development of the questionnaire. We further acknowledge Ronald Isaksen, Administrative Coordinator at SANKS, for his role in organizing and facilitating meetings with youth and schools. Finally, we appreciate the Research Department at Finnmark Hospital Trust for providing a supportive and enabling research environment.”

We note that you have provided funding information that is currently declared in your Funding Statement. However, funding information should not appear in the Acknowledgments section or other areas of your manuscript. We will only publish funding information present in the Funding Statement section of the online submission form.

Please remove any funding-related text from the manuscript and let us know how you would like to update your Funding Statement. Currently, your Funding Statement reads as follows:

“The development of the study protocol was supported by the Research and Development Fund of the Sámi Norwegian National Advisory Unit for Mental Health and Substance Use (SANKS), Finnmark Hospital Trust. The funders had no role in the analysis or interpretation of data, nor did they have any authority over the writing or publication of the manuscript. No additional external funding was received for this study. Funding for the study itself will primarily be provided by Helse Nord, supplemented by institutional research funds from SANKS and Finnmark Hospital Trust. Publication fees and indirect costs will be covered by the hosting institutions.”

Please include your amended statements within your cover letter; we will change the online submission form on your behalf.

Response:

Thank you for your helpful guidance. We have removed all funding-related text from the Acknowledgements section of the manuscript (p. 32), in accordance with your instructions. We confirm that no funding information now appears in any section of the manuscript.

We have revised the Funding Statement accordingly in the online submission form. In doing so, we would like to clarify that the Research and Development Fund of the Sámi Norwegian National Advisory Unit for Mental Health and Substance Use (SANKS) is not an internal institutional fund. Although it is administered by SANKS, it is open to external applicants and involves a competitive review process. The fund is therefore independent of the authors’ institutional affiliation.

As requested, the funding information has been removed from the manuscript and is now provided only in the Funding Statement section of the online submission form. The amended wording has also been included in this cover letter. We kindly ask that the submission system be updated accordingly.

Reviewers' Comments to the Author

Reviewer 1:

This is a clearly written and thoughtfully designed protocol focusing on PIU and positive internet use among Sámi and non-Sámi adolescents in Finnmark. The integration of social norms theory and ethnic identity constructs is well articulated, and the cultural and ethical considerations for Indigenous research are carefully addressed.

Below are several major and minor comments that I believe will strengthen the manuscript and improve its clarity and reproducibility.

Major comments

Comment 1:

Clarify the operationalization of PIU and positive internet use. You state that PIU will be measured using the Problematic Internet Use Questionnaire (PIUQ), and that positive internet use will be captured via items reflecting constructive, identity-supporting, and socially connective internet activities. Please specify explicitly:

(i) Whether the primary PIU outcome in the PLS-SEM models will be a latent factor, a total scale score, or both.

(ii) Whether you plan to use any clinical or empirical cut-off to define “problematic” vs. “non-problematic” internet use (even if only for descriptive purposes), or if PIU will be treated purely as a continuous construct.

(iii) How you will operationalize positive internet use in the structural models (e.g., separate latent factor, composite score, or multiple observed indicators).

Response:

Thank you for your comment. Below, we provide a revised clarification regarding the operationalization of Problematic Internet Use (PIU) and positive internet use, specifically addressing the use of the PIUQ-9 in our study.

(i) Primary PIU Outcome (Latent vs. Total Score)

In the Partial Least Squares Structural Equation Modeling (PLS-SEM) framework, PIU will be modeled as a latent construct with three reflective dimensions: obsession, neglect, and control disorder, as defined by the Problematic Internet Use Questionnaire, Short Form (PIUQ-9). These dimensions are well-supported by the psychometric structure of the PIUQ-9 (Koronczai et al., 2011; Laconi et al., 2019).

For descriptive purposes, we will also calculate and report the total PIUQ-9 score (sum of all 9 items, ranging from 9 to 45). This dual approach allows us to leverage the strengths of both latent variable modeling (for hypothesis testing) and total score reporting (for descriptive comparisons).

This response has been incorporated into measurement of PIU (p.17, l.17–p.18, l.3) in WP1.1: Measuring instruments and Descriptive statistics (p.23, l.3–10) in the Data analysis section.

(ii) Cut-Off vs. Continuous Treatment of PIU

1. Modeling Approach:

In all structural models, PIU will be treated as a continuous latent variable. This approach ensures that the full range of variability in PIU is captured, aligning with the psychometric properties of the PIUQ-9.

2. Descriptive Prevalence Reporting:

For descriptive purposes, we will apply a pre-registered cut-off score of ≥ 22 for the PIUQ-9 (total range: 9–45). This threshold is based on proportional scaling from the original PIUQ-18 cut-off (≥ 41) and aligns with prior psychometric recommendations (Koronczai et al., 2011). While the PIUQ-9 has been validated across diverse cultural contexts, no universally accepted diagnostic cut-off exists (Laconi et al., 2019; Burkauskas et al., 2020). Therefore, the cut-off of ≥ 22 will serve as a descriptive benchmark rather than a diagnostic criterion.

These responses have been reflected into the measurement of PIU (p.18, l.4-13) in WP1.1: Measuring instruments, Descriptive statistics (p.23, l.3–10) and PLS-SEM (p.23, l.12-14) in Data analysis.

(iii) Positive internet use will be operationalized as a separate latent construct in the structural models, as mentioned in the manuscript (maintaining social connections, seeking social support and emotional connection, and engaging with cultural identity). We will conduct a pilot exploratory factor analysis (EFA) during the pretesting phase to confirm the factor structure. If the items load onto a single factor, they will be used as indicators of a unidimensional latent construct. If a multidimensional structure emerges, we will model positive internet use as a higher-order latent construct. Using EFA in the pilot phase ensures the construct is valid and reliable before inclusion in the main study.

This response has been incorporated into the measurement of Positive Internet Use in WP1.1: Measuring instruments (p.18, l.19–p.19, l.6) and PLS-SEM (p.23, l.14-16) in Data analysis.

References

• Koronczai, B., et al. (2011). Confirmation of the three-factor model of problematic internet use on offline adolescent and adult samples. Cyberpsychology, Behavior, and Social Networking, 14(11), 657–664.

• Laconi, S., et al. (2019). Psychometric evaluation of the nine-item Problematic Internet Use Questionnaire (PIUQ-9) in nine European samples of internet users. Frontiers in Psychiatry, 10, 136.

• Burkauskas, J., et al. (2020). Problematic internet use and its relationship with health-related quality of life in a cross-sectional sample: Mediating role of behavioral avoidance. International Journal of Environmental Research and Public Health, 17(22), 8617.

Comment 2:

In your Introduction, you correctly note that PIU is associated with mental health difficulties and some health-risk behaviors, and you reference prior work on PIU in adolescents and young adults. To further strengthen this section, I recommend adding a large epidemiologic study that examined both PIU-substance misuse associations and race/ethnicity-specific prevalence, including Indigenous college students:

Qeadan F, Egbert J, English K. (2022). “Associations between problematic internet use and substance misuse among US college students.” Computers in Human Behavior, 134, 107327.

This study uses data from over 400,000 U.S. college students to quantify the associations between PIU and multiple forms of substance misuse and substance use disorder, and report PIU prevalence across demographic subgroups, including a specific race/ethnicity category for Indigenous students (“NH AI/AN/NH” in Table 1). Including this reference would not only situate your work within the broader PIU-substance use literature for youth and young adults, but also provide readers with complementary prevalence estimates of PIU among Indigenous college students in another national context, which aligns closely with your focus on Sámi adolescents.

Response:

Thank you for this constructive suggestion. We have incorporated the recommended large epidemiologic study by Qeadan, Egbert, and English (2022) into the Introduction under Internet use and Indigenous adolescents (p. 6, l.9-12), and we have added the full citation to the reference list. This addition strengthens our epidemiologic framing of PIU in youth/young adult populations and provides a relevant Indigenous comparison that aligns with our focus on Sámi adolescents.

Comment 3:

The protocol defines Sámi identity based on both objective (ethnic background, language use, ancestry) and subjective (self-identification) criteria, and classifies participants as Sámi if they meet either objective or subjective criteria (or both). Non-Sámi participants are those who report no Sámi ethnic affiliation, language ties, or self-identification as Sámi. Given that your primary analyses will compare Sámi and non-Sámi groups via PLS-MGA, please clarify whether you will:

(i) Distinguish participants who meet only objective criteria from those who meet only subjective criteria, at least in descriptive statistics; and

(ii) Consider sensitivity analyses (e.g., restricting the Sámi group to those meeting both objective and subjective criteria) to assess robustness of any group differences.

Even if your primary analysis retains a single Sámi category, specifying these planned descriptive and sensitivity analyses would help readers appreciate the complexity of Indigenous identity and the potential heterogeneity within the Sámi group.

Response:

We thank the reviewer for raising these important questions regarding the operationalization of Sámi ethnicity. Our approach has been further clarified and refined in response to both the reviewer’s comments and co-author feedback.

In this study, Sámi affiliation is classified using a combination of language use and self-identification, which serve as classification criteria rather than constructs under investigation. This approach is consistent with established practice in Sámi epidemiological and psychosocial research in Norway and has been widely applied in previous large-scale studies, including the North Norwegian Youth Study (NNYS), the Norwegian Arctic Adolescent Health Study (NAAHS), and Ungdata. It is also consistent with broader epidemiological definitions of ethnicity, which conceptualize ethnicity as a multifaceted construct based on “a mix of cultural and other factors including language, ancestry, and self-identity,” and recommend that researchers classify ethnic groups using one or a combination of these indicators (Bhopal, 2004). Using both objective indicators (e.g., language use) and subjective self-identification therefore provides a more comprehensive and theoretically grounded operationalization of Sámi ethnicity.

In addition, to enhance conceptual precision and avoid ambiguity, we have avoided the use of the terms “objective” and “subjective” and instead use the more specific descriptors “language use” and “self-identification,” consistent with established practice in Sámi research (Kvernmo & Heyerdahl, 2004). While the terms “objective” (e.g., ancestry or language use) and “subjective” (self-identification) have been used in some Sámi studies, we follow Kvernmo’s approach by using the descriptors “language use” and “self-identification.” This approach provides clearer and more precise descriptions of the classification criteria applied in the present study.

This combined approach is particularly important in the Norwegian context due to the long history of assimilation policies, which has resulted in discrepancies between ancestry, language use, and self-identification. As shown in previous research (e.g., Kvernmo et al., 2003), a substantial proportion of adolescents with Sámi background may not report Sámi self-identification. Relying solely on one type of indicator would therefore risk systematic misclassification. The primary aim of this study is not to develop new classification methods, but to apply established and comparable criteria to enable robust analysis of group differences.

This terminology has been applied consistently throughout the manuscript (Abstract, p.3; WP1.1: Classification of Sámi and non-Sámi adolescents in WP1.1 Measuring instruments (p.16-17); and PLS Multi Group Analysis (PLS-MGA) in WP3. Data analysis, p.24).

At the same time, we acknowledge that ethnic identity is a multidimensional and dynamic construct, and that no single classification approach can fully capture its complexity. This limitation is explicitly discussed in the revised manuscript (Discussion section, p. 31, l.6-9).

Clarification of group composition

Additionally, we have added a clarification of the group composition to improve transparency:

• The non-Sámi group consists primarily of majority Norwegian adolescents and ethnocultural Kven youth, reflecting the demographic composition of Finnmark. Adolescents from other minority backgrounds are included in this group but are not analyzed separately due to small numbers.

• Adolescents classified as Sámi include individuals with both mono-ethnic and multi-ethnic Sámi backgrounds.

This revision has been incorporated into the section Classification of Sámi and non-Sámi adolescents in WP1.1 Measuring (p.17, l. 8-13).

(i) Distinguishing objective only, subjective only, and combined Sámi identifiers in descriptive statistics

In response to the reviewer’s suggestion, we will provide descriptive statistics differentiating adolescents who meet only language-based criteria, only self-identification criteria, or both. This will allow readers to clearly see the heterogeneity within the Sámi group, which is particularly important in light of assimilation processes that have created discrepanci

Attachment Submitted filename: renamed_c6b9f.docx

# Peer review: Decision Letter 1

**DOI:** 10.1371/journal.pone.0356920.r004

**Authors:** José Perales

7 Jul 2026

Please submit your revised manuscript by Aug 21 2026 11:59PM. If you will need more time than this to complete your revisions, please reply to this message or contact the journal office at plosone@plos.org. When you're ready to submit your revision, log on to https://www.editorialmanager.com/pone/ and select the 'Submissions Needing Revision' folder to locate your manuscript file.

- A letter that responds to each point raised by the academic editor and reviewer(s). You should upload this letter as a separate file labeled 'Response to Reviewers'.
- A marked-up copy of your manuscript that highlights changes made to the original version. You should upload this as a separate file labeled 'Revised Manuscript with Track Changes'.
- An unmarked version of your revised paper without tracked changes. You should upload this as a separate file labeled 'Manuscript'.

If you would like to make changes to your financial disclosure, please include your updated statement in your cover letter. Guidelines for resubmitting your figure files are available below the reviewer comments at the end of this letter.

If applicable, we recommend that you deposit your laboratory protocols in protocols.io to enhance the reproducibility of your results. Protocols.io assigns your protocol its own identifier (DOI) so that it can be cited independently in the future. For instructions see: https://journals.plos.org/plosone/s/submission-guidelines#loc-laboratory-protocols. Additionally, PLOS ONE offers an option for publishing peer-reviewed Lab Protocol articles, which describe protocols hosted on protocols.io. Read more information on sharing protocols at https://plos.org/protocols?utm_medium=editorial-email&utm_source=authorletters&utm_campaign=protocols.

As the corresponding author, your ORCID iD is verified in the submission system and will appear in the published article. PLOS supports the use of ORCID, and we encourage all coauthors to register for an ORCID iD and use it as well. Please encourage your coauthors to verify their ORCID iD within the submission system before final acceptance, as unverified ORCID iDs will not appear in the published article. *Only*  the individual author can complete the verification step; PLOS staff *cannot*  verify ORCID iDs on behalf of authors.

We look forward to receiving your revised manuscript.

Kind regards,

José C. Perales

Academic Editor

PLOS One

Journal Requirements:

If the reviewer comments include a recommendation to cite specific previously published works, please review and evaluate these publications to determine whether they are relevant and should be cited. There is no requirement to cite these works unless the editor has indicated otherwise.

Please review your reference list to ensure that it is complete and correct. If you have cited papers that have been retracted, please include the rationale for doing so in the manuscript text, or remove these references and replace them with relevant current references. Any changes to the reference list should be mentioned in the rebuttal letter that accompanies your revised manuscript. If you need to cite a retracted article, indicate the article’s retracted status in the References list and also include a citation and full reference for the retraction notice.

[Note: HTML markup is below. Please do not edit.]

Reviewers' comments:

Reviewer's Responses to Questions

**Comments to the Author**

1. Does the manuscript provide a valid rationale for the proposed study, with clearly identified and justified research questions?

Reviewer #1: Yes

Reviewer #2: Yes

**********

2. Is the protocol technically sound and planned in a manner that will lead to a meaningful outcome and allow testing the stated hypotheses?

Reviewer #1: Yes

Reviewer #2: Yes

**********

3. Is the methodology feasible and described in sufficient detail to allow the work to be replicable?

Reviewer #1: Yes

Reviewer #2: Yes

**********

4. Have the authors described where all data underlying the findings will be made available when the study is complete?

The PLOS Data policy

Reviewer #1: Yes

Reviewer #2: Yes

**********

5. Is the manuscript presented in an intelligible fashion and written in standard English?

Reviewer #1: Yes

Reviewer #2: Yes

**********

Please use the space provided to explain your answers to the questions above and, if applicable, provide comments about issues authors must address before this protocol can be accepted for publication. You may also include additional comments for the author, including concerns about research or publication ethics.

You may also provide optional suggestions and comments to authors that they might find helpful in planning their study.

Reviewer #1: Thank you for the thorough and constructive revision. The manuscript is much improved, and my substantive concerns have been addressed.

I have no further substantive comments. Before publication, please remove or resolve the residual phrase “rural (?)” in the Discussion. You may also consider adding one brief sentence noting whether measurement invariance across questionnaire language versions will be explored if sample size permits, or otherwise acknowledged as a limitation.

Reviewer #2: I thank the authors for the substantial effort invested in this revision. The manuscript is clearer and several of my previous concerns have been adequately addressed, particularly the distinction between perceived and objective norms, the definition of PIU, and the revised approach to Sámi self-identification.

A few issues still require clarification before the protocol is fully ready. First, the 'positive internet use' construct still needs a clearer theoretical and measurement rationale, including the full items and a pre-specified validation strategy; an EFA in a very small pilot sample would not be sufficient for this purpose.

Second, the proposed cut-off score (≥22) for the PIUQ-9, derived via proportional scaling from the PIUQ-18, lacks psychometric robustness. Even if used strictly for descriptive purposes, this threshold remains methodologically fragile. The authors should either provide a stronger empirical justification for this specific cut-off or explicitly acknowledge this limitation regarding the validity of their prevalence estimates.

**********

what does this mean?). If published, this will include your full peer review and any attached files.

If you choose “no”, your identity will remain anonymous but your review may still be made public.

**Do you want your identity to be public for this peer review?**  For information about this choice, including consent withdrawal, please see our Privacy Policy

Reviewer #1: No

Reviewer #2: **Yes:**  Jose López-Guerrero

**********

[NOTE: If reviewer comments were submitted as an attachment file, they will be attached to this email and accessible via the submission site. Please log into your account, locate the manuscript record, and check for the action link "View Attachments". If this link does not appear, there are no attachment files.]

To ensure your figures meet our technical requirements, please review our figure guidelines: https://journals.plos.org/plosone/s/figures

You may also use PLOS’s free figure tool, NAAS, to help you prepare publication quality figures: https://journals.plos.org/plosone/s/figures#loc-tools-for-figure-preparation.

NAAS will assess whether your figures meet our technical requirements by comparing each figure against our figure specifications.

# Author comment: Author response to Decision Letter 2

**DOI:** 10.1371/journal.pone.0356920.r005

9 Jul 2026

Reviewer 1:

Thank you for the thorough and constructive revision. The manuscript is much improved, and my substantive concerns have been addressed.

I have no further substantive comments. Before publication, please remove or resolve the residual phrase “rural (?)” in the Discussion. You may also consider adding one brief sentence noting whether measurement invariance across questionnaire language versions will be explored if sample size permits, or otherwise acknowledged as a limitation.

Response

Thank you for your positive assessment of the revised manuscript and for your helpful suggestions. The residual phrase “rural (?)” in the Discussion has been corrected and now reads: “The study is relevant for both regional and broader efforts to promote digital well-being among adolescents in Arctic, rural, and Indigenous contexts.” (p. 30, l. 4-5)

Regarding measurement invariance across questionnaire language versions, we have added a statement to the limitations of the Discussion (p.30, l.23 – p.31, l.2). Although the questionnaire has undergone forward–backward translation and cultural adaptation, the number of participants completing the Northern Sámi version may be limited. Consequently, formal assessment of measurement invariance across language versions may not be feasible for all constructs.

Reviewer 2:

I thank the authors for the substantial effort invested in this revision. The manuscript is clearer and several of my previous concerns have been adequately addressed, particularly the distinction between perceived and objective norms, the definition of PIU, and the revised approach to Sámi self-identification.

A few issues still require clarification before the protocol is fully ready. First, the 'positive internet use' construct still needs a clearer theoretical and measurement rationale, including the full items and a pre-specified validation strategy; an EFA in a very small pilot sample would not be sufficient for this purpose.

Second, the proposed cut-off score (≥22) for the PIUQ-9, derived via proportional scaling from the PIUQ-18, lacks psychometric robustness. Even if used strictly for descriptive purposes, this threshold remains methodologically fragile. The authors should either provide a stronger empirical justification for this specific cut-off or explicitly acknowledge this limitation regarding the validity of their prevalence estimates.

Response:

Thank you for your constructive feedback and for recognizing the substantial revisions made to the manuscript.

We agree that the rationale and validation strategy for the positive internet use construct required further clarification. We have revised the manuscript to describe positive internet use as a conceptually and empirically informed construct derived from previous research on Indigenous youth, rather than as an established psychometric scale. This clarification has been incorporated into the WP1.1: Measuring Instruments section of the Methods (p.18, l.19 – p.19, l.11). Previous studies among Indigenous youth in Australia and Canada have shown that digital technologies may promote social connection, emotional support, community belonging, cultural engagement, and identity affirmation (Rice et al., 2016; Castleton, 2018; Johnson, 2016; Paul et al., 2024). Based on this literature, positive internet use is conceptualized as adolescents' use of digital technologies to maintain social connections, seek social and emotional support, and engage with cultural identity. These domains were further refined through interviews with Sámi and non-Sámi adolescents to ensure cultural relevance in the Finnmark context.

We have also revised the validation strategy. Rather than relying on exploratory factor analysis (EFA) in the small pilot sample, the pilot phase will be used primarily to assess item clarity, relevance, and feasibility. The dimensionality, reliability, and construct validity of the positive internet use items will subsequently be evaluated in the full study sample prior to inclusion in the structural models. In line with the study protocol's commitment to transparency, the Norwegian and Northern Sámi versions of the questionnaire will be made openly accessible for research purposes following completion of the study and publication of the results.

Regarding the PIUQ-9 cut-off score, we agree that the proposed threshold (≥22), derived through proportional scaling from the PIUQ-18, has limited psychometric validation. We have therefore revised the manuscript to acknowledge this limitation more explicitly. The manuscript now clarifies that no universally validated cut-off currently exists for the PIUQ-9 and that the threshold of ≥22 will be used solely as a descriptive benchmark rather than as a diagnostic criterion. We have further noted in the limitations in the Discussion section that prevalence estimates based on this threshold should be interpreted with caution due to the limited empirical validation of the cut-off (p.31, l.14-16).

References cited in this response

• Castleton, A. (2018). Technology and Inuit identity: Facebook use by Inuit youth. AlterNative, 14(3), 228–236.

• Johnson, G. M. (2016). Technology use among Indigenous adolescents in remote regions of Australia. International Journal of Adolescence and Youth, 21(2), 218–231.

• Paul, J., McQuaid, R. J., Hopkins, C., Perri, A., Stewart, S., Matheson, K., Anisman, H., & Bombay, A. (2024). Relations between bullying and distress among youth living in First Nations communities: Assessing direct and moderating effects of culture-related variables. Transcultural Psychiatry, 61(3), 429–439.

• Rice, E. S., Haynes, E., Royce, P., & Thompson, S. C. (2016). Social media and digital technology use among Indigenous young people in Australia: A literature review. International Journal for Equity in Health, 15, 81.

Attachment Submitted filename: renamed_54034.docx

# Peer review: Decision Letter 2

**DOI:** 10.1371/journal.pone.0356920.r006

**Authors:** José Perales

12 Aug 2026

Study Protocol: Risk and resilience factors in problematic internet use among Sámi and non-Sámi adolescents in Finnmark, Arctic Norway: the role of social norms and ethnic identity

PONE-D-25-51272R2

Dear Dr. Hansen,

We’re pleased to inform you that your manuscript has been judged scientifically suitable for publication and will be formally accepted for publication once it meets all outstanding technical requirements.

Within one week, you’ll receive an e-mail detailing the required amendments. When these have been addressed, you’ll receive a formal acceptance letter and your manuscript will be scheduled for publication.

An invoice will be generated when your article is formally accepted. Please note, if your institution has a publishing partnership with PLOS and your article meets the relevant criteria, all or part of your publication costs will be covered. Please make sure your user information is up-to-date by logging into Editorial Manager at Editorial Manager® and clicking the ‘Update My Information' link at the top of the page. For questions related to billing, please contact billing support.

If your institution or institutions have a press office, please notify them about your upcoming paper to help maximize its impact. If they’ll be preparing press materials, please inform our press team as soon as possible -- no later than 48 hours after receiving the formal acceptance. Your manuscript will remain under strict press embargo until 2 pm Eastern Time on the date of publication. For more information, please contact onepress@plos.org.

Kind regards,

José C. Perales

Academic Editor

PLOS One

Additional Editor Comments (optional):

Reviewers' comments:

Reviewer's Responses to Questions

**Comments to the Author**

1. Does the manuscript provide a valid rationale for the proposed study, with clearly identified and justified research questions?

Reviewer #2: Yes

**********

2. Is the protocol technically sound and planned in a manner that will lead to a meaningful outcome and allow testing the stated hypotheses?

Reviewer #2: Yes

**********

3. Is the methodology feasible and described in sufficient detail to allow the work to be replicable?

Reviewer #2: Yes

**********

4. Have the authors described where all data underlying the findings will be made available when the study is complete?

The PLOS Data policy

Reviewer #2: Yes

**********

5. Is the manuscript presented in an intelligible fashion and written in standard English?

Reviewer #2: Yes

**********

Please use the space provided to explain your answers to the questions above and, if applicable, provide comments about issues authors must address before this protocol can be accepted for publication. You may also include additional comments for the author, including concerns about research or publication ethics.

You may also provide optional suggestions and comments to authors that they might find helpful in planning their study.

Reviewer #2: I appreciate the authors' considerable effort in revising this manuscript. The corrections introduced satisfactorily address my previous methodological concerns. I wish you the best of luck with the execution of this study.

**********

what does this mean?). If published, this will include your full peer review and any attached files.

If you choose “no”, your identity will remain anonymous but your review may still be made public.

**Do you want your identity to be public for this peer review?**  For information about this choice, including consent withdrawal, please see our Privacy Policy

Reviewer #2: **Yes:**  Jose López-Guerrero

**********

# Editor report: Acceptance letter

**DOI:** 10.1371/journal.pone.0356920.r007

**Authors:** José Perales

PONE-D-25-51272R2

PLOS One

Dear Dr. Hansen,

I'm pleased to inform you that your manuscript has been deemed suitable for publication in PLOS One. Congratulations! Your manuscript is now being handed over to our production team.

At this stage, our production department will prepare your paper for publication. This includes ensuring the following:

* All references, tables, and figures are properly cited

* All relevant supporting information is included in the manuscript submission,

* There are no issues that prevent the paper from being properly typeset

You will receive further instructions from the production team, including instructions on how to review your proof when it is ready. Please keep in mind that we are working through a large volume of accepted articles, so please give us a few days to review your paper and let you know the next and final steps.

Lastly, if your institution or institutions have a press office, please let them know about your upcoming paper now to help maximize its impact. If they'll be preparing press materials, please inform our press team within the next 48 hours. Your manuscript will remain under strict press embargo until 2 pm Eastern Time on the date of publication. For more information, please contact onepress@plos.org.

You will receive an invoice from PLOS for your publication fee after your manuscript has reached the completed accept phase. If you receive an email requesting payment before acceptance or for any other service, this may be a phishing scheme. Learn how to identify phishing emails and protect your accounts at https://explore.plos.org/phishing.

If we can help with anything else, please email us at customercare@plos.org.

Thank you for submitting your work to PLOS One and supporting open access.

Kind regards,

PLOS ONE Editorial Office Staff

on behalf of

Dr. José C. Perales

Academic Editor

PLOS One
